# Bifunctional folic-conjugated aspartic-modified Fe_3_O_4_ nanocarriers for efficient targeted anticancer drug delivery†

**DOI:** 10.1039/d1ra08776b

**Published:** 2022-02-09

**Authors:** Munawar Khalil, Ely Arina Haq, Astari Dwiranti, Eka Sunarwidhi Prasedya, Yoshitaka Kitamoto

**Affiliations:** Department of Chemistry, Faculty of Mathematics and Natural Sciences, Universitas Indonesia 16424 Depok West Java Indonesia mkhalil@sci.ui.ac.id; Department of Biology, Cellular and Molecular Mechanism in Biological System (CEMBIOS) Research Group, Faculty of Mathematics and Natural Sciences, Universitas Indonesia 16424 Depok West Java Indonesia; Department of Biology, Faculty of Mathematics and Natural Sciences, Universitas Mataram 83125 Lombok West Nusa Tenggara Indonesia; Bioscience and Biotechnology Research Center, Faculty of Mathematics and Natural Sciences, University of Mataram 83125 Lombok West Nusa Tenggara Indonesia; Department of Materials Science and Engineering, School of Materials and Chemical Technology, Tokyo Institute of Technology Yokohama 226-8503 Japan

## Abstract

Functionalization of nanocarriers has been considered the most promising way of ensuring an accurate and targeted drug delivery system. This study reports the synthesis of bifunctional folic-conjugated aspartic-modified Fe_3_O_4_ nanocarriers with an excellent ability to deliver doxorubicin (DOX), an anticancer drug, into the intercellular matrix. Here, the presence of amine and carboxylate groups enables aspartic acid (AA) to be used as an efficient anchoring molecule for the conjugation of folic acid (FA) (EDC–NHS coupling) and DOX (electrostatic interaction). Based on the results, surface functionalization showed little effect on the physicochemical properties of the nanoparticles but significantly influenced both the loading and release efficiency of DOX. This is primarily caused by the steric hindrance effect due to large and bulky FA molecules. Furthermore, *in vitro* MTT assay of B16–F1 cell lines revealed that FA conjugation was responsible for a significant increase in the cytotoxicity of DOX-loaded nanocarriers, which was also found to be proportional to AA concentration. This high cytotoxicity resulted from an efficient cellular uptake induced by the over-expressed folate receptors and fast pH triggered DOX release inside the target cell. Here, the lowest IC_50_ value of DOX-loaded nanocarriers was achieved at 2.814 ± 0.449 μg mL^−1^. Besides, further investigation also showed that the drug-loaded nanocarriers exhibited less or no toxicity against normal cells.

## Abstract 2

Aspartic acid was used as an anchoring molecule for the conjugation of folic acid and doxorubicin to Fe_3_O_4_ nanoparticles. The as-prepared bifunctional folic-conjugated aspartic-modified Fe_3_O_4_ nanocarrier was shown to be as an efficient targeted anticancer drug delivery.

## Graphical abstract

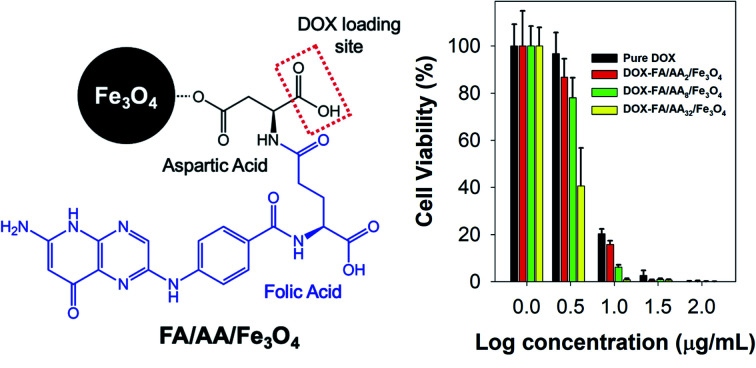

## Introduction

Despite the advancement of medical technologies, cancer is still considered one of the leading contributors to the causes of death globally. Traditionally, cancer is primarily treated with surgery. However, various new strategies, such as radiotherapy, stem cell therapy, chemotherapy, gene therapy, and nanotherapeutics, have recently been developed over several years.^1–6^ Among these techniques, the administration of chemotherapeutic drugs, such as doxorubicin (DOX), has become one of the most promising ways to treat cancer due to their high cytotoxic activity against a wide variety of cancer cells.^7,8^ Nevertheless, several shortcomings often limit an effective DOX administration due to its high cardiotoxicity, low bioavailability and solubility, short bloodstream half-life, and non-specificity.^9–11^ Therefore, current strategies focus on developing a targeted drug delivery system to ensure drug administration at target cancer cells and avoid unnecessary systemic distribution. During the past several years, numerous efforts have been made to develop an efficient and effective anticancer drug carrier. In literature, various types of carries, such as liposomes, micelles, dendrimers, polymeric-based nanoparticles, carbon nanotubes, graphene oxide, and metal/metal oxides nanoparticles, have been reported to exhibit promising abilities in cancer therapy.^2,12–15^

Recently, the application of superparamagnetic Fe_3_O_4_ nanoparticles has gained much attention for biomedical applications due to their low toxicity, high chemical stability, cost-effectiveness, and multifunctionality as new emerging materials for contrasting agents in magnetic resonance imaging (MRI), localized hyperthermia therapy, nanocarrier for drug delivery, or tracking and labelling material in stem cell therapy.^16–20^ For example, Fe_3_O_4_ nanoparticles are often functionalized or composited with other materials such as polymers, small organic molecules, or various other nanomaterials to improve their functionalities. For example, Wang and co-workers reported that encapsulation of graphene oxide-functionalized (Fe_3_O_4_/GO) with folic acid-conjugated chitosan improved loading efficiency of DOX up to 0.98 mg mg^−1^ while still pertaining to the high magnetic saturation of 10.5 emu g^−1^.^21^ It is also demonstrated that the composite material effectively facilitated and efficient pH-trigger drug release due to weakening hydrogen bonds and chitosan degradation. In another study, Karimi and Namazi successfully fabricated and utilized a multifunctional Fe_3_O_4_@PEG-coated dendrimer modified with GO to efficiently deliver DOX.^7^ Based on the *in vitro* results, it was reported that the nanocomposite exhibited a high cellular uptake percentage and demonstrated excellent ability in inducing the apoptosis of breast cancer cells (MCF-17) while maintaining biocompatibility against normal cell line (MCF-10A). Very recently, we have also successfully synthesized and utilized carboxylates functionalized Fe_3_O_4_ nanoparticles for efficient loading and release DOX in chemotherapy of HeLa (cervical cancer) cell lines.^8^ Based on the investigation, we demonstrated that different carboxylate moieties played a crucial role in dictating the ability of Fe_3_O_4_ nanoparticles in both DOX loading and pH-controlled release. The result showed that functionalization of the nanoparticles with citric acid exhibited the highest efficiency in inducing the death of HeLa cells due to the strong interaction between DOX and citrate residue at the surface of Fe_3_O_4_ nanoparticles.

Additionally, conjugation of drug-loaded Fe_3_O_4_ nanoparticles with a specific ligand that can selectively recognize cancer cell targets has also been widely studied for targeted delivery vehicles. Among various types of these ligands, folic acid (FA) has received much attention since folate receptors are known to be selectively overexpressed at a wide variety of cancer cells, such as brain, skin, breast, kidney, and lung.^21^ In addition, it is also due to its low molecular weight and high binding affinity (*K*_d_ = 1 × 10^−10^ M).^22,23^ Therefore, the combination of external targeting strategy by a guided magnetic field and FA conjugation are expected to enhance the ability of Fe_3_O_4_-based nanocarriers to precisely deliver the loaded drug to target cells. For instance, Yang and co-workers successfully conjugated FA to Fe_3_O_4_ nanoparticles loaded with diblock copolymers of poly(ethylene glycol) PEG and poly(ε-caprolactone) PCL for efficient delivery of anticancer drug.^24^ Based on the result, the attachment of FA onto polymer micelles was responsible for the specific recognition of the drug-loaded nanocarrier to reach the cancer cell target, which was indicated by the high cellular uptake. Furthermore, FA-conjugated iron-modified multiwalled carbon nanotubes have also been reported to exhibit excellent ability as targeted DOX nanocarrier to induce the apoptosis of HeLa cells.^25^ Here, it was reported that the nanocarriers showed a high DOX loading capacity (32 μg mg^−1^) and prolonged release capability triggered by external near-infrared radiation.

Nevertheless, most of the current FA conjugation involves utilizing large and bulky anchoring molecules, such as polymers or carbon-based materials, or separate moieties in addition to the one for DOX. Consequently, the presence of these multiple conjugating and anchoring molecules for FA and the drug would limit the optimum drug loading capacity and reduce the magnetization of Fe_3_O_4_ nanoparticles. Therefore, this study reports the utilization of bifunctional aspartic acid (AA) to develop efficient targeted Fe_3_O_4_ nanoparticles-based nanocarriers with high anticancer drug loading capacity. In this study, AA was selected due to the presence of the amine (NH_2_) group as the anchoring site for FA and two carboxylate (COOH) groups for the conjugating sites of DOX and the surface of the nanoparticles. Besides, the utilization of AA was also due to its high biocompatibility as one of the essential amino acids. Here, the efficiency of the as-prepared bifunctional folic-conjugated aspartic-modified Fe_3_O_4_ nanoparticles (FA/AA/Fe_3_O_4_) in loading and pH-triggered release of DOX was studied at various concentrations of AA (2, 8, and 32 mmol). For convenience, the as-prepared samples were denoted as AA_2_/Fe_2_O_3_, AA_8_/Fe_3_O_4_, and AA_32_/Fe_3_O_4_ for Fe_3_O_4_ nanoparticles modified with 2, 8, and 32 mmol of AA, respectively. Furthermore, the performance of the nanocarriers in cancer therapy was evaluated against the B16–F1 cell line (human skin cancer).

## Materials and methods

### Materials

Iron(ii) chloride tetrahydrate (FeCl_2_·4H_2_O) (purity: 97%), iron(iii) chloride hexahydrate (FeCl_3_·6H_2_O) (purity: 98%), ammonium hydroxide (NH_4_OH) solution (28–30% of NH_3_ in H_2_O) (purity: >93%), l-aspartic acid (C_4_H_7_NO_4_) (purity: 98.5%) were used in the synthesis of aspartic-modified Fe_3_O_4_ nanoparticles. Furthermore, folic acid (FA) (C_19_H_19_N_7_O_6_) (purity: 97%), *N*-(3-dimethylaminopropyl)-*N*′-ethylcarbodiimide hydrochloride (EDC) (C_8_H_17_N_3_·HCl) (purity: 98%), and *N*-hydroxysuccinimide (NHS) (C_4_H_5_NO_3_) (purity: 98%) were used in the conjugation of FA. Besides, doxorubicin hydrochloride (DOX) was supplied by an Indonesian pharmaceutical company (PT. Kalbe Farma, Tbk) and used as the anticancer drug. Moreover, hydrochloric acid (HCl 37%) and sodium hydroxide (NaOH) were used to adjust the pH of the solution. Meanwhile, sodium acetate (CH_3_COONa) (purity: 99%), acetic acid (CH_3_COOH) (purity: 99.7%), dipotassium phosphate (K_2_HPO_4_) (purity: 99%), and monopotassium phosphate (KH_2_PO_4_) (purity: 99%) were used to prepare acetate buffer pH 5 and phosphate buffer saline (PBS) pH 7.2. All chemicals were in analytical grade and used without additional purification.

### Synthesis of aspartic-modified Fe_3_O_4_ nanoparticles

Aspartic-modified Fe_3_O_4_ nanoparticles were prepared using the co-precipitation method according to our previous works.^8,26,27^ Typically, 0.87 g of FeCl_2_·4H_2_O and 2.22 g of FeCl_3_·6H_2_O were diluted in 40 mL of deionized water. Afterward, the mixture was then mixed using a magnetic stirrer for 30 minutes at 70 °C. Subsequently, 5 mL of NH_4_OH was slowly added into the mixture while further stirred for another 30 minutes. Furthermore, an aqueous solution of l-aspartic acid was then added into the mixture at various concentrations, *i.e.*, 2, 8, and 32 mmol. The mixture was then let to further react for another hour at 90 °C under ambient atmospheric pressure. After the reaction, the resulting black precipitate was then collected using an external magnet and washed with deionized water and ethanol, respectively. Finally, the resulting powder was then dried in a vacuum oven for 24 hours and used for further investigations. For comparison, unmodified Fe_3_O_4_ nanoparticles were prepared using the same method in a separate reaction without adding l-aspartic acid.

### Synthesis of folic-conjugated aspartic-modified Fe_3_O_4_ nanoparticles

In this work, an EDC–NHS coupling reaction was used to conjugate FA onto the as-prepared Fe_3_O_4_ nanoparticles modified with various amounts of AA according to the work reported by Rana and co-workers.^28^ Typically, the conjugation of FA was carried out by dissolving 5 mg of FA (0.1 mg mL^−1^) in deionized water and placed in an ultrasonic bath for 15 minutes to improve the dissolution process. Afterward, 0.5 mL (1 mg mL^−1^) of NHS and 0.5 mL (1 mg mL^−1^) of EDC were added into the mixture while further ultrasonicated for another 20 minutes to activate the carboxylate group in FA. Subsequently, the mixture was added into a colloidal dispersion of 84 mg of the as-prepared AA/Fe_3_O_4_ in 20 mL of deionized water. The mixture was then let to react for 3 hours in continuous stirring using a magnetic stirrer. Finally, the precipitate was collected using an external magnet and washed with deionized water and ethanol. The resulting powder was then dried in a vacuum oven for 24 hours and used for further characterizations.

### Characterizations

Several characterization methods were employed to evaluate the physicochemical properties of the as-prepared nanoparticles samples. In this study, the crystalline phases of the samples were studied using X-ray diffraction (XRD) analysis using PANanalytical X'Pert Pro MPD (PANanalytical B.V., Amelo, the Netherlands), where Cu-Kα (*λ* = 1.5406 Å) was used as the X-ray source. Meanwhile, micrographic and selected area electron diffraction (SAED) analyses of the as-prepared nanoparticles were also carried out using TECNAI G2 Spirit Twin High-Resolution Transmission Electron Microscope (HRTEM) (operational voltage of 200 kV). Here, the analysis was carried out by adding few drops of colloidal nanoparticles sample into PELCO^®^ 200 mesh copper grid (Ted Pella, Inc.). The estimation of particle size was done by collecting the size of some 300–500 particles using a virtual ruler (ImageJ). In addition, the magnetic properties of the powder samples were also analyzed using Lake Shore 7400 Series Vibration Sample Magnetometer (VSM) (Lake Shore Cryotronic, Inc., Ohio) at room temperature with a maximum field of 8000 Oe. Fourier-transform infrared (FTIR) spectroscopy analysis was also conducted to study the functional groups of the samples using Shimadzu IRPrestige-21 FTIR spectrophotometer. Here, the analysis was carried out to a pellet sample prepared by mixing a small amount of the nanoparticle samples with KBr. Thermogravimetric analysis (TGA) was also carried out to study aspartic acid functionalization using TGA Q500 (TA Instrument) with nitrogen (N_2_) gas at a temperature range of 25 to 800 °C and a heating rate of 10 °C minute^−1^. Besides, Horiba SZ-100 (Horiba Scientific) was also used to measure the zeta potential (*ζ*) of the samples. Finally, a Shimadzu UV-2450 spectrophotometer was also used to study the conjugation of FA onto the nanoparticle samples.

### Doxorubicin (DOX) loading and release

The ability of the as-prepared nanoparticles as nanocarriers of the anticancer drug was evaluated by carrying out DOX loading and release efficiency according to our previous report.^8^ In this work, DOX loading was conducted by preparing the as-prepared FA/AA/Fe_3_O_4_ samples at various concentrations, *i.e.*, 20, 60, 100, and 140 μg mL^−1^ in deionized water. An equal amount of each colloidal dispersion was then mixed with 10 μg mL^−1^ of DOX while subjected to ultrasonication for 30 minutes at room temperature. After the reaction, the corresponding DOX-loaded FA/AA/Fe_3_O_4_ sample (DOX-FA/AA/Fe_3_O_4_) was then collected using an external magnet. Meanwhile, the supernatant was then used to determine loading efficiency using Thermo Fischer Scientific Varioskan LUX Multimode Microplate Reader at excitation and emission wavelength of 490 and 535 nm. Here, the DOX loading efficiency (%) was then estimated by comparing the fluorescence intensity of DOX in the supernatant and the initial DOX solution, which can be calculated as follows:1
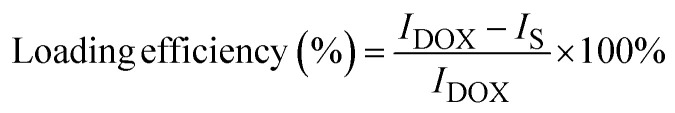
where, *I*_DOX_ and *I*_S_ represent the fluorescence intensity of DOX in the supernatant and the initial DOX solution, respectively.

Meanwhile, the efficiency of DOX release was evaluated *via* the dialysis method. Typically, 1 mL of the as-prepared FA/AA/Fe_3_O_4_ colloidal suspension (10 mg mL^−1^) was vigorously mixed with 2 mL of an aqueous solution of DOX (2 mg mL^−1^) for an hour at dark. Subsequently, DOX-loaded nanoparticles were then collected using an external magnet. Furthermore, 10 mg of the collected nanoparticles were redispersed in 5 mL of acetate buffer pH. The dispersion was then placed in a dialysis bag and the dialysis was carried out using 200 mL of phosphate buffer saline (PBS) pH 7.4 at 37 °C for 36 hours. During the dialysis, an aliquot was collected every 6 hours, and the amount of released DOX was determined using Thermo Fischer Scientific Varioskan LUX Multimode Microplate Reader at excitation and emission wavelength of 490 and 535 nm, where the overall DOX release efficiency (%) can be estimated according to the following equation:2
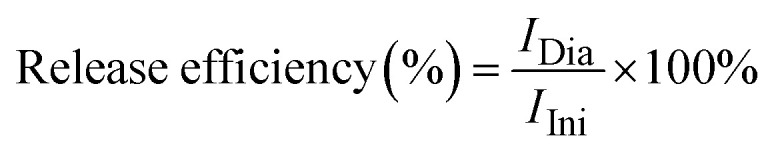
Here, *I*_Dia_ is the fluorescence intensity of the solution after dialysis. Meanwhile, *I*_Ini_ represents the fluorescence intensity of the initial sample solution before dialysis.

### Cell culture

B16–F1 cell line was purchased from ECACC cell lines Sigma-Aldrich. Cells were cultured in DMEM (Gibco, Scotland) in a 75 cm^2^ tissue flask (Nunc, Denmark) and passaged every 2–3 days after trypsinization with trypsin/EDTA. Each 500 mL was supplemented with 10% Fetal Bovine Serum (FBS), 10 mL penicillin/streptomycin (50 IU/50 μg mL^−1^), 10 mL of sodium pyruvate (1 mM), NaHCO_3_ (2 g) and 10 mL of l-glutamine (2 mM). The supplemented medium was then filtered using 0.22 μm microfilters and stored at 4 °C before use.

### Cytotoxicity assay

The cytotoxic effects of pure DOX and DOX-loaded nanocarriers were evaluated *in vitro* on B16–F1 cells with a rapid colorimetric assay using MTT (Biovision, USA). This assay is based on the metabolic reduction of soluble MTT by mitochondrial enzyme activity of viable tumor cells into an insoluble colored formazan product, which can be measured spectrophotometrically after dissolving in DMSO. A volume of 100 μL cell suspension (3 × 10^4^ cells per mL) was dispensed into 96-well microplates (Nunc, Denmark) and incubated at 37 °C in a fully humidified atmosphere of 5% CO_2_. After 24 hours, the medium was changed with serial dilutions of DOX-AA/FA/Fe_3_O_4_ colloidal solutions (1–100 μg mL^−1^). Evaluation of cell survival was done in 24 h treatment of cells with the as-prepared DOX-AA/FA/Fe_3_O_4_ colloidal solutions. Treatment mediums were changed with 100 μL MTT reagent (5 mg mL^−1^ in PBS) for 3 hours. Then the medium was replaced with 150 μL DMSO and complete solubilization of formazan crystals was achieved by repeated pipetting of the solution. Absorbance was then determined at 590 nm by an ELISA plate reader (Multiskan GO, Thermo Scientific). The cytotoxic effect was expressed as the relative viability and calculated as shown below. Relative viability = (sample absorbance − blank)/(absorbance of untreated controls − blank) × 100%. The non-linear regression dose–response curve and IC_50_ value were calculated with Graphpad Prism (Graphpad Inc., San Diego, CA, USA). For comparison, a similar cytotoxicity assay was also carried out using pure DOX solution and DOX-AA/Fe_3_O_4_ samples.

### Cell viability evaluation with fluorescence imaging

Cells were stained with the fluorescent probes calcein–AM and propidium iodide (PI). Because of the permeable ability of the cell membrane, calcein–AM was used to stain viable cells, while PI was used to label dead cells. B16–F1 cells were plated onto 35 mm^2^ culture dishes and cultured overnight to achieve cell adhesion. Then cells were treated with 100 μg mL^−1^ concentrations of pure DOX and DOX-AA/FA/Fe_3_O_4_ colloidal solutions. After 24 h of treatment, the cells were washed three times with PBS, followed by incubation with a PBS solution mixed with 2 μL calcein-AM (10 mg mL^−1^) at 37 °C for 15 minutes. Finally, cells were stained with 2 μL PI (10 mg mL^−1^) before visualization under Zeiss Axio Observer 7 Inverted Fluorescence Microscope (Zeiss, Germany). Additionally, B16–F10 murine melanoma cells (ECACC 92101204) and NIH-3T3 normal murine fibroblasts (ECACC 93061524) were purchased from the European Collection of Authenticated Cell Cultures. The cells were routinely cultivated in Dulbecco's modified Eagle medium (DMEM, Wako) supplemented with 10% FBS for B16–F10 and 5% for NIH-3T3. The cells were kept at 37 °C in a 5% CO_2_ humidified incubator (Forma Stericycle, Thermofisher Scientific). For all experiments, cells were grown in T-25 cell culture flasks with a seeding density of 0.8 × 10^6^ cells per mL. After reaching 80–90% confluence, cells were then seeded according to the experimental needs.

### Statistical analysis

All the analysis was carried out based on one-way analysis of variance (ANOVA) and *T*-test comparison with significant levels of *p* < 0.05. Each experiment was performed in triplicates, and all data were presented as mean ± standard deviation.

## Results and discussion

### Fabrication of aspartic-modified Fe_3_O_4_ nanoparticles

**Fig. 1:**
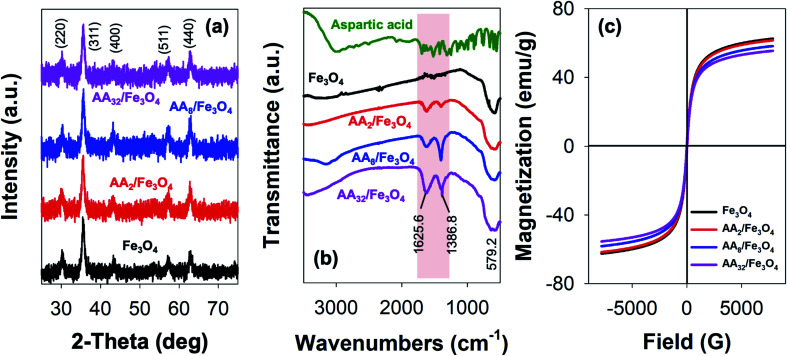
(a) XRD pattern, (b) FTIR spectra, and (c) magnetization curves of unmodified Fe_3_O_4_ and the corresponding aspartic-modified Fe_3_O_4_ nanoparticles.

Fig. 1a presents the result obtained from the XRD analysis. Based on the outcome, it is evident that all reaction products could be unambiguously indexed as magnetite (Fe_3_O_4_). This is primarily due to the appearance of characteristic Bragg's peaks for the inverse spinel crystal phase, which was found to be in good agreement with the database (JCPDS card no. 88-0315) and those reported elsewhere.^2,7,8,26–29^ Besides, the result also demonstrated that the formation of such crystal phase and their degree of crystallinity was not affected by surface modification with aspartic acid. As shown, both unmodified and aspartic-modified Fe_3_O_4_ exhibited a very similar XRD pattern. This observation was also accurate for the modification of Fe_3_O_4_ nanoparticles with various amounts of aspartic acid.

Furthermore, the reaction products were also characterized by FTIR spectroscopy. Here, the formation of magnetite crystal and the successful attachment of aspartic acid onto the surface of Fe_3_O_4_ could be evaluated by analyzing the resulting IR peaks. Fig. 1b shows FTIR spectra of aspartic acid, unmodified Fe_3_O_4_, and aspartic-modified Fe_3_O_4_ nanoparticles. As shown, the formation of magnetite was further supported by the appearance of a characteristic peak at 579.2 cm^−1^ for the stretching vibration of the Fe–O bond at magnetite crystal lattice.^30–32^ This peak was observed at unmodified and aspartic-modified Fe_3_O_4_ nanoparticles. However, modification with aspartic acid resulted in two new sharp peaks at 1625.6 and 1386.8 cm^−1^, which were absent at the unmodified Fe_3_O_4_ spectrum. Interestingly, it is also worth noting that the intensity of such peaks was also proportional to the concentration of aspartic acid. It is believed that these peaks were originated from stretching vibration of symmetric (*ν*_s_) and asymmetric (*ν*_as_) of COO– due to the attachment of aspartic acid on the surface of Fe_3_O_4_. In literature, the coordination mode between carboxylate head (COO–) and Fe atoms at the iron oxide crystals could be determined by calculating the wavenumber separation (Δ*ν*) between *ν*_s_ and *ν*_as_.^33^ Zhang and co-workers reported that the interaction mode can be monodentate (Δ*ν* = 200–300 cm^−1^), bridging bidentate (Δ*ν* = 140–190 cm^−1^), or chelating bidentate (Δ*ν* < 110 cm^−1^).^34^ Therefore, the result revealed that the coordination mode between aspartic acid could be classified as monodentate interaction since the estimated Δ*ν* was found to be 238.8 cm^−1^. Moreover, this successful surface modification was also supported by TGA analysis, where the attached aspartic acid moieties were detached at high temperatures (see Fig. S1a, ESI†). Additionally, the result demonstrated that the as-prepared nanoparticles exhibited excellent colloidal stability, especially at the physiological condition. This is indicated by the negative values of their zeta potential (*ζ*) (Fig. S1b, ESI†). In literature, studies have reported that nanoparticles with good colloidal stability tend to exhibit *ζ* value larger than 25 mV or lower than −25 mV.^35,36^ Besides, it is also worth noting that the colloidal stability of the aspartic-modified Fe_3_O_4_ nanoparticles seems to be increasing with the amount of attached aspartic acid.

In addition, VSM measurements revealed that the as-prepared unmodified and aspartic-modified Fe_3_O_4_ nanoparticles exhibited superparamagnetism. This is indicated by the appearance of a typical symmetrical sigmoidal magnetization curve with a lack of hysteresis loop (Fig. 1c).^37^ Based on the domain theory, superparamagnetism behavior is obtained when the grain size of the nanoparticles is smaller than ∼30 nm, which is the diameter of zero-coercivity (*D*_p_).^38–40^ Consequently, the magnetic domain of the nanomaterial can be transformed from multidomain (MD) to single domain (SD), resulting in a large coercivity since all of the magnetic spins are aligned in the same direction.^41^ Based on the result, the maximum magnetization (*M*_s_) value for the as-prepared unmodified Fe_3_O_4_ nanoparticles was found to be 62.5 emu g^−1^ (Fig. 1c), which was larger than the same Fe_3_O_4_ nanoparticles prepared by co-precipitation (60.5 emu g^−1^) and hydrothermal (43.2 emu g^−1^) reported elsewhere.^42,43^ Nevertheless, it is worth mentioning that modification with aspartic acid resulted in a slight reduction of *M*_s_. Besides, the magnitude of such reduction was proportional with the amount of aspartic acid. As shown in Fig. 1c, the resulting *M*_s_ values for AA_2_/Fe_3_O_4_, AA_8_/Fe_3_O_4_, AA_32_/Fe_3_O_4_ were found to be 61.6, 58.2, and 55.4 emu g^−1^, respectively. This excellent magnetic property was also proven by the efficient magnetic sedimentation of the nanoparticles under the influence of an external magnetic field (Fig. S2, ESI†). Therefore, the as-prepared nanoparticles hold great potential for efficient anticancer drug delivery and as MRI contrast agents and hyperthermia treatment.

**Fig. 2:**
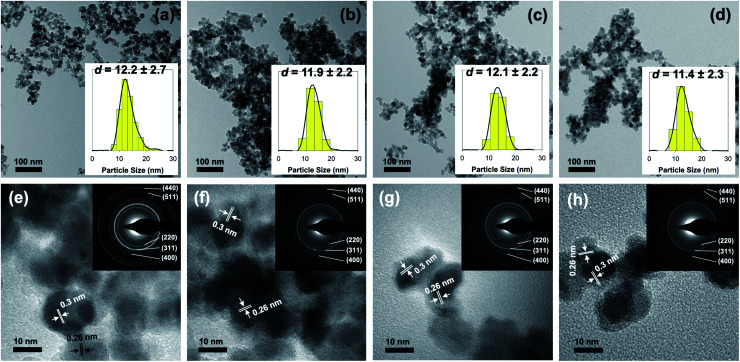
(a–d) TEM (inset: particle size distributions) and (e–h) HR-TEM images (inset: SAED patterns) of unmodified Fe_3_O_4_ and the corresponding AA_2_/Fe_3_O_4_, AA_8_/Fe_3_O_4_, AA_32_/Fe_3_O_4_ AA_32_ nanoparticles, respectively.

Further investigation using a transmission electron microscope was also carried to determine the influence of surface modification in the morphology and size of the as-prepared nanoparticles. Fig. 2 presents the result obtained from both TEM and HR-TEM analyses. Based on the result, it is evident that the co-precipitation method was able to make highly monodisperse Fe_3_O_4_ nanoparticles with spherical-like morphology. Additionally, it is revealed that surface modification with aspartic acid did not lead to a significant change in either size or morphology of the as-prepared nanoparticles (Fig. 2a–d). As shown, particle size estimation showed that the average diameter of unmodified Fe_3_O_4_, AA_2_/Fe_3_O_4_, AA_8_/Fe_3_O_4_, and AA_32_/Fe_3_O_4_ nanoparticles were found to be 12.2 ± 2.7, 11.9 ± 2.2, 12.1 ± 2.2, and 11.4 ± 2.3 nm, respectively. Moreover, additional evidence for the formation of magnetite inverse spinel crystal phase was also obtained from HR-TEM analysis. This is due to the appearance of characteristic lattice fringes of magnetite's (311) and (220) crystal planes at 0.26 and 0.3 nm, respectively (Fig. 2e–h). In addition, the selected area electron diffraction (SAED) analysis demonstrated that the obtained ring patterns were in good agreement with the literature and the result from XRD analysis (inset of Fig. 2e–h). This observation was accurate for both unmodified and aspartic-modified Fe_3_O_4_ nanoparticles.

### Conjugation with folic acid

**Fig. 3:**
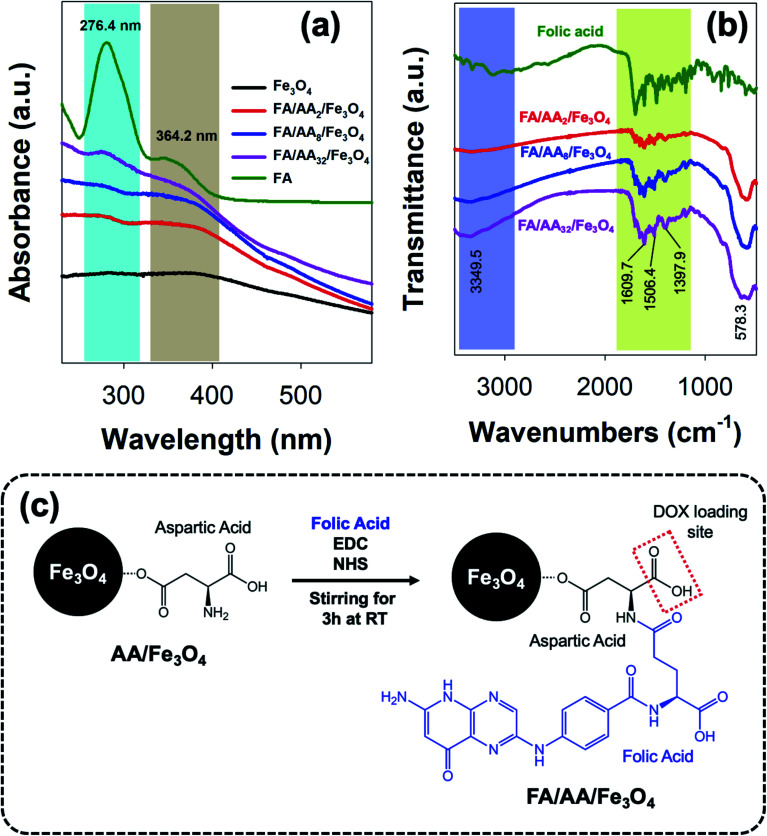
(a) UV-Vis and (b) FTIR spectra of the as-prepared folic-conjugated aspartic-modified Fe_3_O_4_ nanoparticles (FA/AA/Fe_3_O_4_); (c) schematic illustration of the conjugation of folic acid *via* EDC–NHS coupling reaction.

As previously mentioned, further functionalization of the as-prepared aspartic-modified Fe_3_O_4_ with folic acid (FA) was carried out to enable site-specific targeting for DOX delivery. Here, the conjugation of FA was done *via* EDC–NHS coupling reaction and evaluated using UV-Vis and FTIR spectroscopies. The result shows that the as-prepared AA/Fe_3_O_4_ nanoparticles were successfully functionalized with FA due to the formation of an amide bond between the EDC–NHS activated carboxylic acid group at FA amine group at AA. This successful FA attachment is indicated by the appearance of characteristic absorption peaks at 276.4 and 364.2 nm due to π → π* transition of the pterin ring and n → π* transition of the *p*-amino benzoyl acid (PABA), respectively, which were also clearly observed at the spectrum of pristine FA (Fig. 3a).^44,45^ Interestingly, it is also noticeable that the intensity of these peaks was increased with the amount of AA used for the modification of Fe_3_O_4_. This suggests that the amount of attached FA is proportional to the concentration of AA at the nanoparticles' surface.

In addition, a similar observation was also revealed by FTIR analysis. As shown in Fig. 3b, it is evident that FA was successfully conjugated to AA/Fe_3_O_4_ nanoparticles. This is proven by the similar resemblance of the as-prepared FA/AA/Fe_3_O_4_ nanoparticles spectrum to the pristine folic acid, especially at the fingerprint region. Besides, this is also supported by the appearance of several characteristic peaks belongs to functional groups of FA at FA/AA/Fe_3_O_4_ spectrum. For example, the appearance of sharp peaks at 1397.9, 1506.4, and 1609.7 cm^−1^ could be ascribed due to IR absorption of the phenyl ring, N–H bending vibration, and C=O amide stretching of the α-carboxyl group, respectively.^28,46^ Besides, broadening of N–H vibration at 3349.5 cm^−1^ was also believed to be originated from the formation of the amide bond, suggesting the successful linkage of FA to AA/Fe_3_O_4_ nanoparticles. Overall, the schematic illustration for the functionalization of AA/Fe_3_O_4_ nanoparticles with FA *via* EDC–NHS coupling reaction is presented in Fig. 3c.

### Loading and release of DOX

**Fig. 4:**
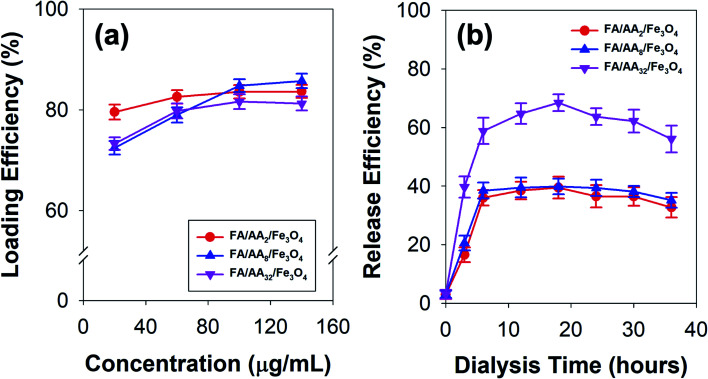
(a) Doxorubicin (DOX) loading efficiency at various concentrations of the as-prepared nanocarriers, and (b) time-dependent release efficiency of DOX-loaded nanocarriers.

To further investigate the applicability of the as-prepared FA/AA/Fe_3_O_4_ in drug delivery systems, doxorubicin's loading and release efficiency (DOX) were also studied. Fig. 4a presents the obtained loading efficiency of DOX at various concentrations of nanocarriers. Based on the result, it is evident that DOX has been successfully loaded onto the as-prepared nanoparticles with considerably high loading efficiency. This is true since DOX is widely known to exhibit a strong affinity to negatively charged functional groups, such as phospholipids, carboxylates, and oleate.^28,47–49^ Therefore, the observed high loading efficiency to FA/AA/Fe_3_O_4_ could be originated from the ability of DOX to form a strong electrostatic interaction with carboxylate moiety of aspartic acid. It is believed that this interaction resulted from partial protonation of DOX's amine group and deprotonation of aspartic acid's carboxylate group of the nanocarrier at low pH, which was indicated by the negative value of *ζ*-potential (see Fig. S1, ESI†). Additionally, we also confirmed that self-quenching of DOX due to the π–π stacking was not the case since the fluorescence intensity of pure DOX in the absence of nanoparticles was not changed over time (see Fig. S3, ESI†).

Interestingly, the result also demonstrated that the loading efficiency of DOX increased with the concentration of all types of nanocarriers. This is expected since more nanocarriers were available for DOX loading. Nevertheless, it is worth noting that the behavior of DOX conjugation was also affected by the concentration of aspartic acid used to modify nanoparticles (Fig. 4a). At low concentration (40 μg mL^−1^), result showed that FA/AA_2_/Fe_3_O_4_ exhibited a significantly higher loading efficiency (79.52%) than that of FA/AA_8_/Fe_3_O_4_ (72.45%) and FA/AA_32_/Fe_3_O_4_ (73.27%). Meanwhile, the efficiency was very similar when the loading was carried out at a high concentration. It is believed that such observation could be attributed to the steric hindrance effect resulting from FA conjugation. The higher the concentration of aspartic acid for modification, the higher the amount of FA that could be conjugated. As a result, this would lead to a blockage and more restricted electrostatic interaction between the deprotonated carboxyl group of aspartic acid and the protonated amine group of DOX, which ultimately caused the lower DOX loading efficiency.

Furthermore, Fig. 4b shows the time-dependent release behavior of DOX loaded onto a different type of nanocarriers. Here, DOX release efficiency was performed by mimicking the cell environment condition where pH 5 buffer acetate and pH 7.4 PBS were used as reservoir and sink, respectively. Based on the result, it is evident that DOX was quickly released within the first 6 h of the dialysis and gradually reached equilibrium after 12 h (Fig. 4b). This immediate release was believed to be caused by the weakening of electrostatic interaction between DOX and the nanocarriers, which was stimulated by the decrease of pH. Moreover, the result also revealed that FA/AA_32_/Fe_3_O_4_ exhibited a significantly higher DOX release efficiency than that of FA/AA_2_/Fe_3_O_4_ and FA/AA_8_/Fe_3_O_4_ nanocarriers. Such a phenomenon was expected since the interaction between DOX and FA/AA_32_/Fe_3_O_4_ was disrupted by the steric hindrance effect of FA. Consequently, DOX could easily be detached from FA/AA_32_/Fe_3_O_4_ when subjected to the typical acidic cancer cell environment, ultimately leading to a higher DOX release efficiency (Fig. 4b). Interestingly, it is also worth noting that FA/AA_2_/Fe_3_O_4_ and FA/AA_8_/Fe_3_O_4_ nanocarriers showed no big difference in release behavior. Such observation is believed to be caused by the fact the two nanocarriers exhibited a very similar loading efficiency (Fig. 4a).

### *In vitro* cytotoxicity of B16–F1 cells

**Fig. 5:**
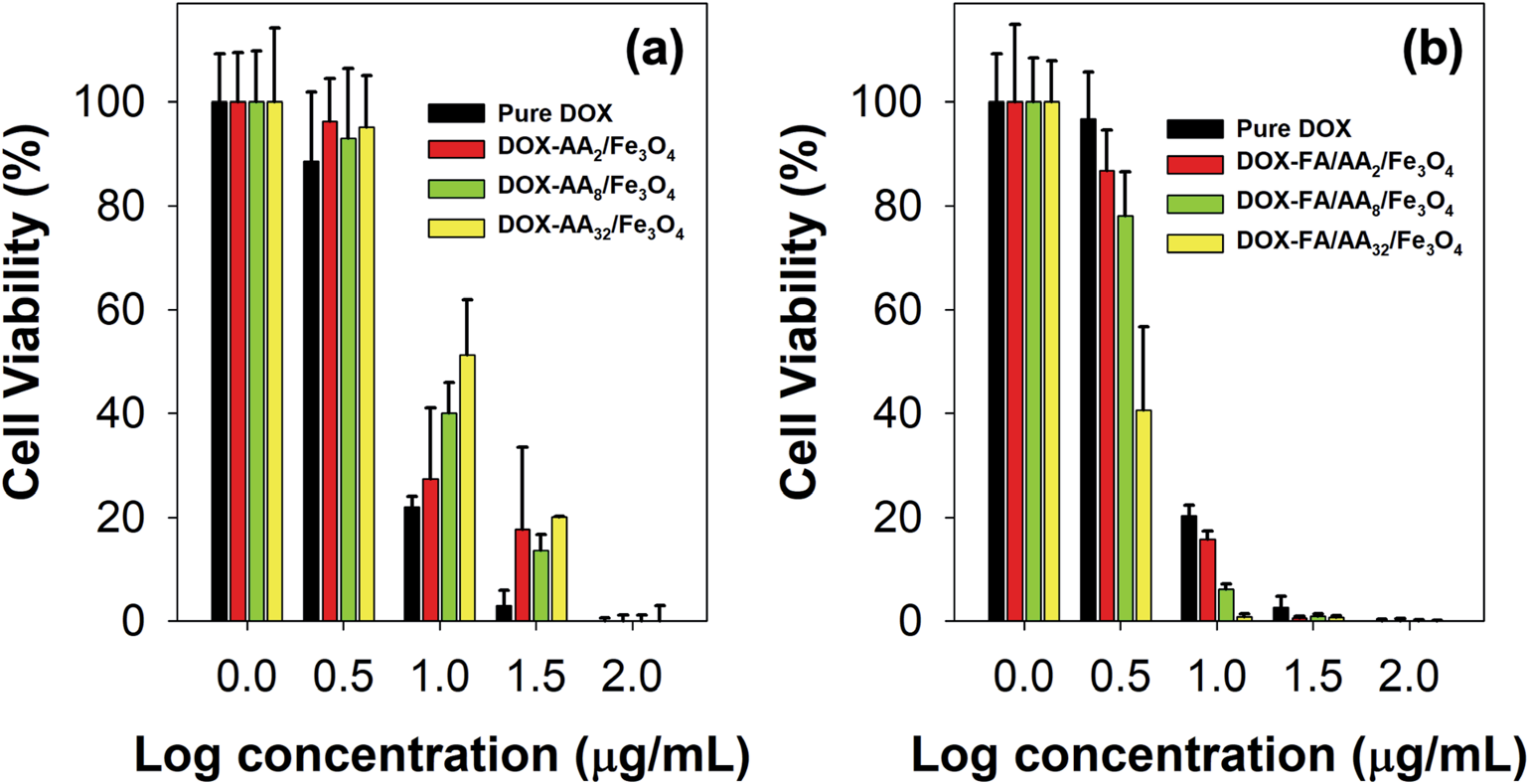
B16–F1 cell viability against different concentrations of (a) pure DOX and DOX-loaded AA/Fe_3_O_4_ (nanocarriers without FA), and (b) pure DOX and DOX-loaded FA/AA/Fe_3_O_4_ (nanocarriers with FA) (all experiments were carried out in triplicates).

To further evaluate the ability of the as-prepared nanocarriers in targeted delivery of DOX for cancer therapy, MTT assays were also carried out against B16–F1 cell lines incubated with both pure DOX and DOX-loaded nanocarriers. Fig. 5 presents B16–F1 cell viability obtained from MTT assay after *in vitro* chemotherapy. This result is also supported by the estimated values of their IC_50_ (Table 1). Based on the outcome, it is evident both pure DOX and DOX-loaded nanocarriers were cytotoxic towards the human skin cancer cell model. This is indicated by the significant reduction of cell viability with either DOX or DOX-loaded nanocarriers concentration increment. In literature, such toxicity can be related to the ability of DOX in DNA intercalation and inhibition of topoisomerase II.^50–52^ In addition, many studies have also linked the cytotoxicity of DOX with its role in destroying AMP-activated protein kinase and creatine kinase, which are responsible for cellular energy transfer and signaling systems, hence disrupting mitochondrial function.^52^ Besides, it is also reported that the DOX could undergo a one-electron reduction reaction to form semiquinone radicals, producing other reactive oxygen species (ROS), such as superoxide radical anions, and cause the death of cancer cells.^53,54^

**Table 1:** IC_50_ pure DOX and different types of DOX-loaded nanocarriers against B16–F1^a^

Anticancer drug	IC_50_ (μg mL^−1^)
**Without FA conjugation**	
Pure DOX	6.325 ± 2.723
DOX/AA_2_/Fe_3_O_4_	7.329 ± 2.771
DOX/AA_8_/Fe_3_O_4_	8.721 ± 2.001
DOX/AA_32_/Fe_3_O_4_	10.92 ± 1.833

**With FA conjugation**	
Pure DOX	6.120 ± 2.904
DOX/FA/AA_2_/Fe_3_O_4_	5.673 ± 2.964
DOX/FA/AA_8_/ Fe_3_O_4_	4.390 ± 1.328
DOX/FA/AA_32_/Fe_3_O_4_	2.814 ± 0.449

a All experiment were carried out in triplicates.

However, it is also noticed that pure DOX exhibited more cytotoxicity than DOX-AA/Fe_3_O_4_, especially at low concentrations (Fig. 5a). A similar phenomenon was observed in our previous work when DOX was loaded onto Fe_3_O_4_ nanoparticles functionalized with various carboxylate molecules, *i.e.*, succinic acid, ascorbic acid, and citric acid.^8^ It is believed that such observation was primarily due to the ability of free DOX to quickly enter the cell–matrix and intercalate with the cell's DNA *via* simple passive diffusion.^55^ Meanwhile, DOX-AA/Fe_3_O_4_ must penetrate the cell and release the DOX in the lysosome when subjected to the acidity of cancer's intracellular environment. In literature, studies have reported several common mechanisms on how nanoparticles could enter the cell, such as *via* pinocytosis, phagocytosis, endocytosis with clathrin or caveolin-mediated pathway, or clathrin or clathrin or caveolin independent endocytosis.^56–58^ Consequently, this would potentially reduce the amount of DOX delivered into the cell and used for the DNA intercalation. However, it is worth noting that such discrepancy was not the case at high concentrations (100 μg mL^−1^) (Fig. 5a). In addition, the result also demonstrated that the IC_50_ value of DOX/AA/Fe_3_O_4_ seems to be proportional to the concentration of aspartic acid (Table 1). According to the result, it is evident that DOX loaded onto AA_32_/Fe_3_O_4_ nanocarriers exhibited the least cytotoxicity, followed by AA_8_/Fe_3_O_4_ and AA_2_/Fe_3_O_4_, respectively.

Interestingly, FA conjugation was responsible for a significant increment in their ability to deliver DOX and induce cell death. As shown in Fig. 5b, the result revealed that the cytotoxicity of DOX-FA/AA/Fe_3_O_4_ was found to be significantly higher than that of pure DOX. This is also indicated by the low value of their IC_50_, suggesting the superiority of their ability to efficiently increase the cytotoxicity towards B16–F1 cells after incubation for 24 h, regardless of their concentrations (Table 1). Similar enhancement in cellular uptake was also reported by Rana and co-workers when folic acid conjugated Fe_3_O_4_ nanoparticles were used to deliver DOX to KB cells.^28^ It is believed that such remarkable cytotoxicity was primarily due to the enhancement in targeting efficiency of the DOX-loaded nanocarriers. This is expected since FA has a significantly high binding affinity to the over-expressed folate receptors at the cancer cells. Thereby, conjugation of FA would facilitate a more efficient cellular uptake and deliver more DOX for the therapy.

**Fig. 6:**
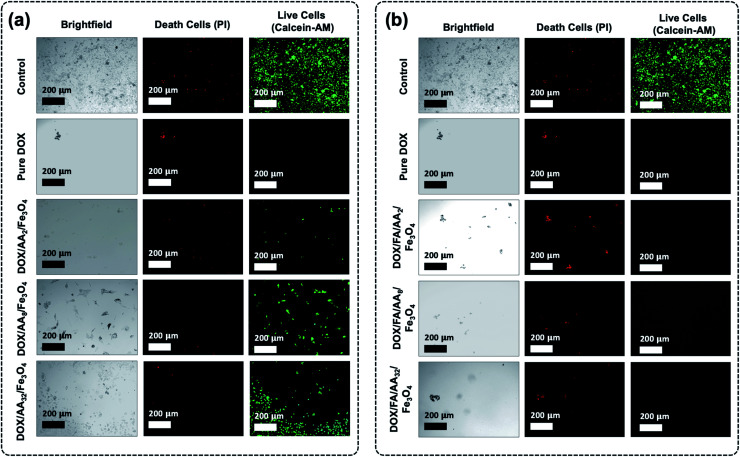
Fluorescence microscopy images of B16–F1 cells before and after incubation of (a) pure DOX and DOX/AA/Fe_3_O_4_, and (b) pure DOX and DOX/FA/AA/Fe_3_O_4_ (death and live cells staining were carried out using PI and calcein–AM).

Furthermore, it is also revealed that the cytotoxicity of the FA conjugated nanocarriers was also influenced by the amount of aspartic acid used in the modification of Fe_3_O_4_ nanoparticles. According to the result obtained from the MTT assay, the ability of the nanocarriers to increase cytotoxicity to B16–F1 cells was found to be proportional to the concentration of aspartic acid (Fig. 5b). This is also supported by the estimated value of their IC_50_. The result shows that the highest cytotoxicity was achieved when DOX was loaded onto FA/AA_32_/Fe_3_O_4_ nanocarriers (Table 1). It is believed that two major reasons caused such observation. One is due to the high amount of conjugated FA at the nanocarriers, enabling an efficient cellular uptake induced by the over-expressed folate receptors. Another reason must be their high DOX release efficiency than FA/AA_2_/Fe_3_O_4_ and FA/AA_8_/Fe_3_O_4_ (see Fig. 4b). It is expected that DOX/FA/AA_32_/Fe_3_O_4_ exhibit an efficient and accurate ability to penetrate the target cancer cells and quickly deliver and release enough DOX into the lysosome. This was also supported by the result obtained from fluorescence microscopy imaging, where both death and live cells could be observed under the influence of different types of DOX-loaded nanocarriers (Fig. 6).

Additionally, cytotoxicity tests with fluorescence microscopy imaging analysis of the as-prepared nanocarriers were also carried out towards normal cells (NIH-3T3 normal murine fibroblasts) (see ESI, Fig. S4 and S5†). Based on the result, it is evident that the as-prepared drug-loaded nanocarriers have significantly less or even no toxicity toward noncancerous normal cells than that observed against B16–F1 when treated at each of their IC_50_ concentration values (ESI, Fig. S4†). Furthermore, similar observation was also obtained when the NIH-3T3 normal cell was treated with drug-loaded nanocarriers at slightly higher concentration (20 μg mL^−1^) (ESI, Fig. S5†). Even then, all drug-loaded nanocarriers showed less toxicity, except DOX/FA/AA_32_/Fe_3_O_4_. This suggests that the utilization of such a system could significantly enhance the delivery of anticancer drugs into the target cancer cell.

## Conclusions

In summary, we have successfully prepared bifunctional folic-conjugated aspartic-modified Fe_3_O_4_ nanocarriers for efficient, targeted delivery of DOX for chemotherapy of B16–F1 (human skin cancer) cells. Here, aspartic acid was successfully used as a molecular anchor for both FA and DOX conjugations. This is primarily due to the presence of the amine group, which was used for FA conjugation *via* EDC–NHS coupling reaction, and the carboxylate group, which was utilized as DOX loading site through the formation of electrostatic interaction at low pH. The result showed that surface modification had none or a very minimal effect on the crystal structure, particle size, colloidal stability, and magnetic properties of the as-prepared Fe_3_O_4_ nanoparticles. However, it is found that both the loading and release efficiency of DOX were highly affected by the amount of AA used in the surface modification and FA conjugation. Results demonstrated that the loading efficiency tends to decrease with the increase of AA concentration, while the opposite effect was observed in the release efficiency. This phenomenon was believed to be primarily caused by the steric hindrance effect due to the presence of large and bulky FA molecules. Furthermore, the MTT assay also revealed that FA conjugation was responsible for significantly enhancing the cytotoxicity of DOX-loaded nanocarriers. Besides, this improvement was also found to be proportional to the concentration of AA. Based on the result, the lowest IC_50_ value could be achieved when DOX was loaded into FA/AA_32_/Fe_3_O_4_ (2.814 ± 0.449 μg mL^−1^), which was significantly lower than that of pure DOX (IC_50_ = 6.120 ± 2.904 μg mL^−1^). Such phenomenon was believed to be the result of an efficient cellular uptake induced by the over-expressed folate receptors and the ability of the nanocarriers to quickly release the loaded drug inside the intracellular matrix when subjected to the typical acidic cancer cell environment. In addition, results also demonstrated that the drug-loaded nanocarriers exhibited less or no toxicity against normal cell.

## Author contributions

M. K. is responsible for conceiving and developing the idea, funding acquisition, data analysis, performed the experiments, results and discussion, writing and editing the manuscript; E. A. H., and E. S. P. performed the experiments and data analysis; A. D. and Y. K. involved in funding acquisition and data analysis.

## Conflicts of interest

The authors declare no competing interest in this study.

## Supplementary Material

RA-012-D1RA08776B-s001

## References

[cit1] Huang R.-X., Zhou P.-K. (2020). Signal Transduction Targeted Ther..

[cit2] Karthika V., AlSalhi M. S., Devanesan S., Gopinath K., Arumugam A., Govindarajan M. (2020). Sci. Rep..

[cit3] Mirza Z., Karim S. (2021). Semin. Cancer Biol..

[cit4] Shen S., Xu X., Lin S., Zhang Y., Liu H., Zhang C., Mo R. (2021). Nat. Nanotechnol..

[cit5] Yang L., Shi P., Zhao G., Xu J., Peng W., Zhang J., Zhang G., Wang X., Dong Z., Chen F. (2020). Signal Transduction Targeted Ther..

[cit6] Zhu Y., Fang Y., Kaskel S. (2010). J. Phys. Chem. C.

[cit7] Karimi S., Namazi H. (2021). J. Alloys Compd..

[cit8] Saepudin E., Fadhilah H. R., Khalil M. (2020). Colloids Surf., A.

[cit9] Duan Q., Ma Y., Che M., Zhang B., Zhang Y., Li Y., Zhang W., Sang S. (2019). J. Drug Delivery Sci. Technol..

[cit10] Toomari Y., Namazi H., Entezami A. A. (2015). Int. J. Biol. Macromol..

[cit11] Yang C., Wu T., Qin Y., Qi Y., Sun Y., Kong M., Jiang X., Qin X., Shen Y., Zhang Z. (2018). Int. J. Nanomed..

[cit12] Jiang L., Zhou S., Zhang X., Li C., Ji S., Mao H., Jiang X. (2021). Nat. Commun..

[cit13] Sheveleva N. N., Markelov D. A., Vovk M. A., Mikhailova M. E., Tarasenko I. I., Neelov I. M., Lähderanta E. (2018). Sci. Rep..

[cit14] Zhang C.-g., Zhu W.-j., Liu Y., Yuan Z.-q., Chen W.-l., Li J.-z., Zhou X.-f., Liu C., Zhang X.-n. (2016). Sci. Rep..

[cit15] Thapa B., Diaz-Diestra D., Santiago-Medina C., Kumar N., Tu K., Beltran-Huarac J., Jadwisienczak W. M., Weiner B. R., Morell G. (2018). ACS Appl. Bio Mater..

[cit16] Barick K. C., Singh S., Jadhav N. V., Bahadur D., Pandey B. N., Hassan P. A. (2012). Adv. Funct. Mater..

[cit17] Chandra S., Dietrich S., Lang H., Bahadur D. (2011). J. Mater. Chem..

[cit18] Gazeau F., Lévy M., Wilhelm C. (2008). Nanomedicine.

[cit19] Popescu R. C., Savu D., Dorobantu I., Vasile B. S., Hosser H., Boldeiu A., Temelie M., Straticiuc M., Iancu D. A., Andronescu E., Wenz F., Giordano F. A., Herskind C., Veldwijk M. R. (2020). Sci. Rep..

[cit20] Popescu R. C., Savu D. I., Bierbaum M., Grbenicek A., Schneider F., Hosser H., Vasile B. S., Andronescu E., Wenz F., Giordano F. A., Herskind C., Veldwijk M. R. (2021). Int. J. Mol. Sci..

[cit21] Wang Z., Zhou C., Xia J., Via B., Xia Y., Zhang F., Li Y., Xia L. (2013). Colloids Surf., B.

[cit22] Campbell I. G., Jones T. A., Foulkes W. D., Trowsdale J. (1991). Cancer Res..

[cit23] Weitman S. (1992). Distribution of the folate receptor GP38 in normal and malignant cell lines and tissues. Cancer Res..

[cit24] Yang X., Chen Y., Yuan R., Chen G., Blanco E., Gao J., Shuai X. (2008). Polymer.

[cit25] Li R., Wu R. A., Zhao L., Hu Z., Guo S., Pan X., Zou H. (2011). Carbon.

[cit26] Khalil M., Aulia G., Budianto E., Mohamed Jan B., Habib S. H., Amir Z., Abdul Patah M. F. (2019). ACS Omega.

[cit27] Khalil M., Fahmi A., Nizardo N. M., Amir Z., Mohamed Jan B. (2021). Langmuir.

[cit28] Rana S., Shetake N. G., Barick K. C., Pandey B. N., Salunke H. G., Hassan P. A. (2016). Dalton Trans..

[cit29] Sutariya S., Bsatee M., Gololobova O., Diaz-Diestra D., Thapa B., Weiner B. R., Morell G., Jadwisienczak W. M., Beltran-Huarac J. (2021). ACS Omega.

[cit30] Eivazzadeh-Keihan R., Maleki A. (2021). J. Nanostruct. Chem..

[cit31] Fadhilah H. R., Saepudin E., Khalil M. (2020). AIP Conf. Proc..

[cit32] Zhou J., Wang L., Qiao X., Binks B. P., Sun K. (2012). J. Colloid Interface Sci..

[cit33] Khalil M., Yu J., Liu N., Lee R. L. (2014). Colloids Surf., A.

[cit34] Zhang L., He R., Gu H.-C. (2006). Appl. Surf. Sci..

[cit35] Tekade R. K., Tekade M., Kumar M., Chauhan A. S. (2015). Pharm. Res..

[cit36] Wang P., Keller A. A. (2009). Langmuir.

[cit37] Savva I., Marinica O., Papatryfonos C. A., Vekas L., Krasia-Christoforou T. (2015). RSC Adv..

[cit38] Khalil M., Jan B. M., Tong C. W., Berawi M. A. (2017). Appl. Energy.

[cit39] Samrot A. V., Sahithya C. S., Selvarani A J., Purayil S. K., Ponnaiah P. (2021). Current Research in Green and Sustainable Chemistry.

[cit40] Zhi D., Yang T., Yang J., Fu S., Zhang S. (2020). Acta Biomater..

[cit41] Issa B., Obaidat I. M., Albiss B. A., Haik Y. (2013). Int. J. Mol. Sci..

[cit42] Barick K. C., Aslam M., Lin Y.-P., Bahadur D., Prasad P. V., Dravid V. P. (2009). J. Mater. Chem..

[cit43] Zhang Y.-Q., Wei X.-W., Yu R. (2010). Catal. Lett..

[cit44] Baibarac M., Smaranda I., Nila A., Serbschi C. (2019). Sci. Rep..

[cit45] Chakraborty P., Bairi P., Roy B., Nandi A. K. (2014). ACS Appl. Mater. Interfaces.

[cit46] Zhang Y., Kohler N., Zhang M. (2002). Biomaterials.

[cit47] De Wolf F. A., Nicolay K., De Kruijff B. (1992). Biochemistry.

[cit48] Munnier E., Tewes F., Cohen-Jonathan S., Linassier C., Douziech-Eyrolles L., Marchais H., Soucé M., Hervé K., Dubois P., Chourpa I. (2007). Chem. Pharm. Bull..

[cit49] Nigam S., Barick K. C., Bahadur D. (2011). J. Magn. Magn. Mater..

[cit50] Greco F., Vicent M. J., Gee S., Jones A. T., Gee J., Nicholson R. I., Duncan R. (2007). J. Controlled Release.

[cit51] Slovak M. L., Hoeltge G. A., Dalton W. S., Trent J. M. (1988). Cancer Res..

[cit52] Tomankova K., Polakova K., Pizova K., Binder S., Havrdova M., Kolarova M., Kriegova E., Zapletalova J., Malina L., Horakova J. (2015). Int. J. Nanomed..

[cit53] Kuznetsov A. V., Margreiter R., Amberger A., Saks V., Grimm M. (2011). Biochim. Biophys. Acta, Mol. Cell Res..

[cit54] Lüpertz R., Wätjen W., Kahl R., Chovolou Y. (2010). Toxicology.

[cit55] Kamba S. A., Ismail M., Hussein-Al-Ali S. H., Ibrahim T. A. T., Zakaria Z. A. B. (2013). Molecules.

[cit56] Kamaly N., Miller A. D. (2010). Int. J. Mol. Sci..

[cit57] Modo M. M. and Bulte J. W., Molecular and Cellular MR Imaging, CRC Press, 2007, pp. 1–9

[cit58] Mukherjee S., Ghosh R. N., Maxfield F. R. (1997). Physiol. Rev..

