# Supplementary material for: Bifunctional folic-conjugated aspartic-modified Fe3O4 nanocarriers for efficient targeted anticancer drug delivery
Source: RSC Adv. 2022 Feb 9;12(8):4961–71. doi: 10.1039/d1ra08776b (PMC8981417; doi:10.1039/d1ra08776b)
Supplement: RA-012-D1RA08776B-s001 [file RA-012-D1RA08776B-s001.pdf]

Electronic Supplementary Material (ESI) for RSC Advances.
This journal is © The Royal Society of Chemistry 2022

Bifunctional folic-conjugated aspartic-modified Fe_3O_4 nanocarriers for efficient targeted anticancer drug delivery

Munawar Khalil[1]*, Ely Arina Haq[1], Astari Dwiranti[2], Eka Sunarwidhi Prasedya[3,4], Yoshitaka Kitamoto[5]

[1]Department of Chemistry, Faculty of Mathematics and Natural Sciences, Universitas Indonesia, 16424 Depok, West Java, Indonesia.
[2]Cellular and Molecular Mechanism in Biological System (CEMBIOS) Research Group, Department of Biology, Faculty of Mathematics and Natural Sciences, Universitas Indonesia, 16424 Depok, West Java, Indonesia
[3]Department of Biology, Faculty of Mathematics and Natural Sciences, Universitas Mataram, 83125 Lombok, West Nusa Tenggara, Indonesia.
[4]Bioscience and Biotechnology Research Center, Faculty of Mathematics and Natural Sciences, University of Mataram, 83125 Lombok, West Nusa Tenggara, Indonesia.
[5]Department of Materials Science and Engineering, School of Materials and Chemical Technology, Tokyo Institute of Technology, Yokohama 226-8503, Japan.

*Corresponding author: mkhalil@sci.ui.ac.id

Supplementary Information

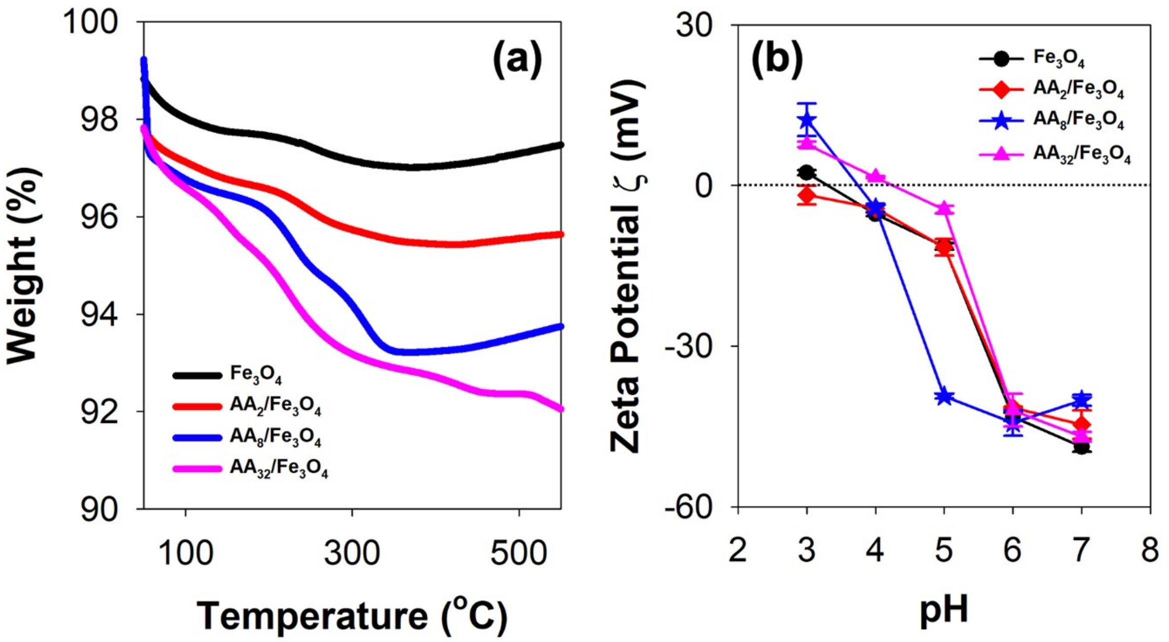

Figure S1. (a) TGA curves, and (b) zeta potential (ζ) values at various pH for the unmodified Fe_3O_4 and the corresponding Fe_3O_4/AA_2, Fe_3O_4/AA_8, and Fe_3O_4/AA_{32} nanoparticles, respectively.

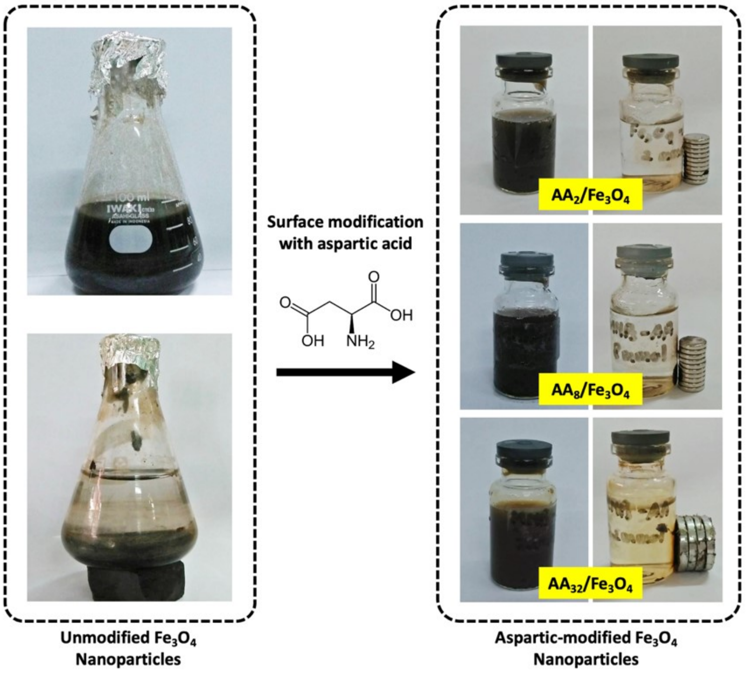

Figure S2. Photograph of unmodified and aspartic-modified Fe_3O_4 without and with the influence of external permanent magnet.

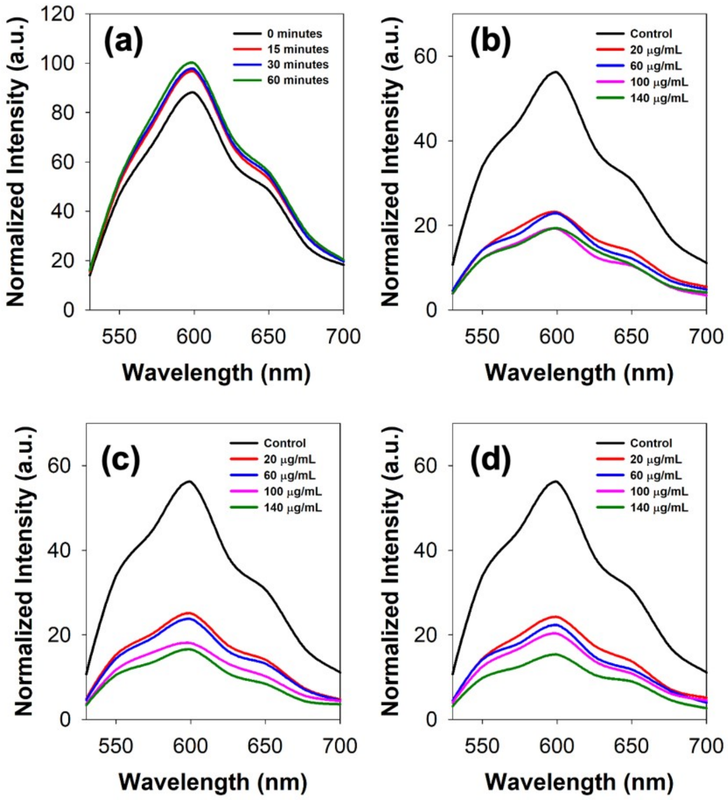

Figure S3. Normalized fluorescence spectra of (a) pure DOX at different time intervals, and (b-d) DOX after loading at various concentrations of $FA/AA_2/Fe_3O_4$, $FA/AA_8/Fe_3O_4$, and $FA/AA_{32}/Fe_3O_4$ nanoparticles, respectively.

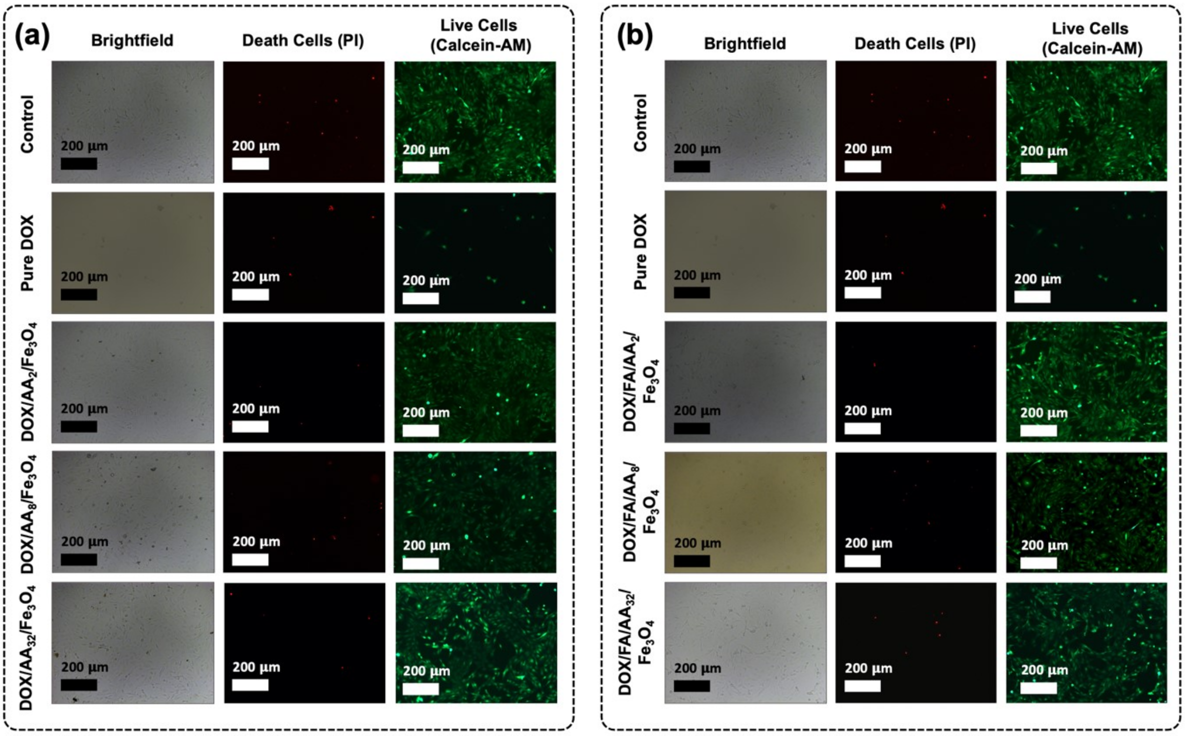

Figure S4. Fluorescence microscopy images of NIH-3T3 normal murine fibroblasts before and after incubation of (a) pure DOX and DOX/AA/Fe_3O_4, and (b) pure DOX and DOX/FA/AA/Fe_3O_4 at their IC_{50} concentration.

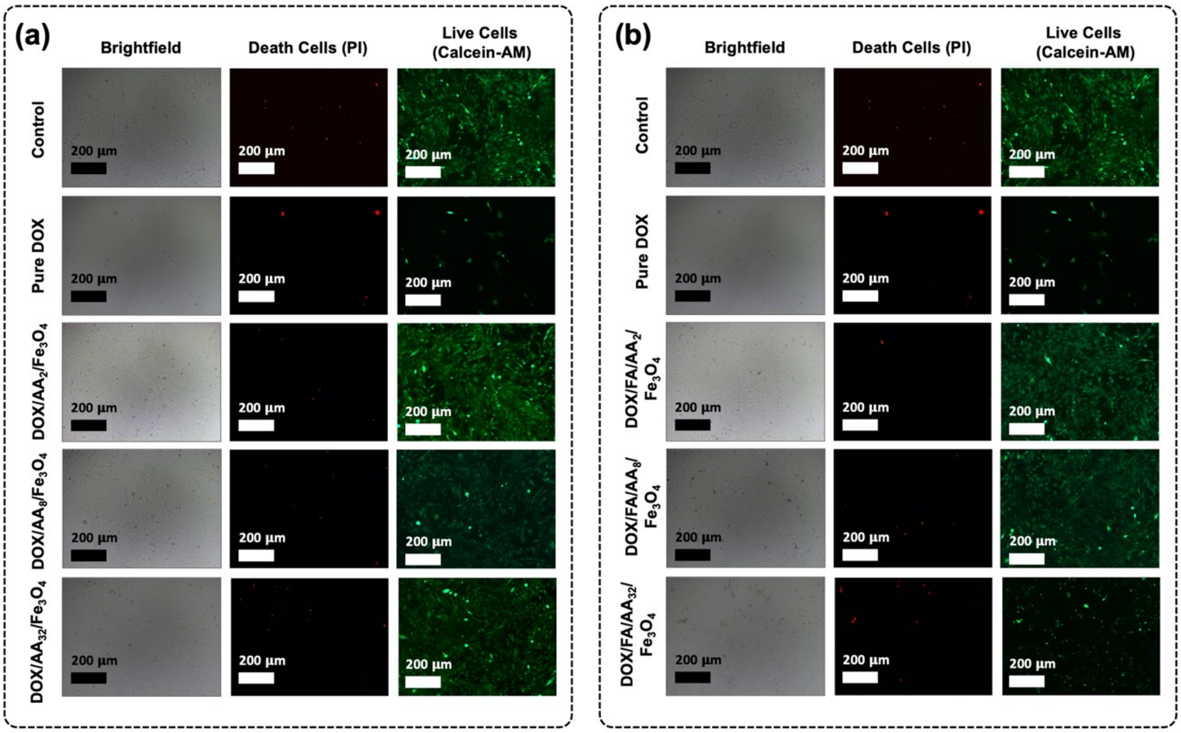

Figure S5. Fluorescence microscopy images of NIH-3T3 normal murine fibroblasts before and after incubation of (a) pure DOX and DOX/AA/Fe_3O_4, and (b) pure DOX and DOX/FA/AA/Fe_3O_4 at concentration of 20 µg/mL.
